# Evaluation of Parkinson Disease Risk Variants as Expression-QTLs

**DOI:** 10.1371/journal.pone.0046199

**Published:** 2012-10-05

**Authors:** Jeanne C. Latourelle, Alexandra Dumitriu, Tiffany C. Hadzi, Thomas G. Beach, Richard H. Myers

**Affiliations:** 1 Department of Neurology, Boston University School of Medicine, Boston, Massachusetts, United States of America; 2 Bioinformatics Program, Boston University College of Arts and Sciences, Boston, Massachusetts, United States of America; 3 Civin Laboratory for Neuropathology, Banner Sun Health Research Institute, Sun City, Arizona, United States of America; UCL Institute of Neurology, United Kingdom

## Abstract

The recent Parkinson Disease GWAS Consortium meta-analysis and replication study reports association at several previously confirmed risk loci *SNCA*, *MAPT*, *GAK/DGKQ*, and *HLA* and identified a novel risk locus at *RIT2*. To further explore functional consequences of these associations, we investigated modification of gene expression in prefrontal cortex brain samples of pathologically confirmed PD cases (N = 26) and controls (N = 24) by 67 associated SNPs in these 5 loci. Association between the eSNPs and expression was evaluated using a 2-degrees of freedom test of both association and difference in association between cases and controls, adjusted for relevant covariates. SNPs at each of the 5 loci were tested for *cis*-acting effects on all probes within 250 kb of each locus. *Trans*-effects of the SNPs on the 39,122 probes passing all QC on the microarray were also examined. From the analysis of *cis*-acting SNP effects, several SNPs in the *MAPT* region show significant association to multiple nearby probes, including two strongly correlated probes targeting the gene *LOC644246* and the duplicated genes *LRRC37A* and *LRRC37A2*, and a third uncorrelated probe targeting the gene *DCAKD*. Significant *cis*-associations were also observed between SNPs and two probes targeting genes in the HLA region on chromosome 6. Expanding the association study to examine *trans* effects revealed an additional 23 SNP-probe associations reaching statistical significance (p<2.8×10^−8^) including SNPs from the *SNCA, MAPT* and *RIT2* regions. These findings provide additional context for the interpretation of PD associated SNPs identified in recent GWAS as well as potential insight into the mechanisms underlying the observed SNP associations.

## Introduction

Genome-wide association studies (GWAS) have been successful in implicating multiple risk loci that have provided new insights into the complex genetic etiology of Parkinson disease [1]–[9]. The recent meta-analysis and replication study by the US Parkinson Disease GWAS Consortium [10] further strengthened the evidence for association of five previously reported risk loci near the genes for alpha-synuclein (*SNCA*), microtubule associated protein tau (*MAPT*), cyclin G-associated kinase (*GAK*), beta-glucocerebrosidase (*GBA*), and the major histocompatibility complex locus (HLA) and identified a novel risk locus near the gene Ras-like without CAAX 2 (*RIT2*).

However, with the exception of *SNCA* and *GBA*, the GWAS signals do not necessarily specify which gene at the implicated locus is associated with PD risk and none of these findings represents coding variants. The inability of GWAS to pinpoint the specific gene responsible for disease risk is particularly problematic when the region implicated is very large and contains many credible candidates as is the case for the *MAPT* region in PD GWAS [11], [12]. The implicated HLA locus is gene rich and there are no prior candidates to direct future studies.

The determination of the relationship of PD associated SNPs to gene expression levels, offers the potential to highlight the responsible gene, as well as to provide insight into the possible pathological mechanisms associated with these SNPs. In this study, we examine the association between SNPs observed to be strongly associated to PD risk in the US Parkinson Disease GWAS Consortium [10] and gene expression levels in 26 PD and 24 control cortical brain samples [13]. Compellingly, this study not only examines SNP-expression relationships in brain tissue, but specifically in samples with the disease, a strategy which may be necessary for the identification of the disease-related eQTLs [14].

## Results

As part of the US PD-GWAS Consortium meta-analysis replication, all samples were genotyped for all of the top priority association results from the consortium discovery meta-analysis [10]. Six regions (*SNCA*, *MAPT*, *GAK*, *GBA*, HLA, and *RIT2*) were identified to show genome-wide significant association to PD. We identified all SNPs from these six regions that replicated the discovery set's association to PD at a nominal level of significance (p<0.05) [10] resulting in a final set of 67 SNPs, from five different loci (all SNPs genotyped for *GBA* were known coding mutations and these were removed because of low MAF). Several loci, including the *SNCA* and *GAK* regions on chromosome 4, and the *MAPT* region on chromosome 17, included many SNPs in strong to moderate linkage disequilibrium (LD).

Association between the SNPs and cortical gene expression levels from the Agilent 60-mer Whole Human Genome Microarray were evaluated under a dominant model using a 2-degree of freedom (df) linear regression model to simultaneously test association between genotype and expression and difference in association between the 26 PD cases and 24 controls (adjusting for RNA integrity number (RIN), postmortem interval, and age at death, shown in Table 1).

**Table 1:** Brain Sample Characteristics.

	N	Age at death	PMI*	RIN
		(mean ± Stdev)	(mean ± Stdev)	(mean ± Stdev)
PD Cases	26	77.31±8.14	6.57±6.98	7.37±0.89
Controls	24	75.67±11.73	13.26±10.86	7.37±0.86

* *significantly different between cases and controls (p = 0.02).*

### *Cis*-Acting Effects

The initial focus was to evaluate *cis*-acting effects of the PD associated SNPs. The 67 SNPs from the five loci were tested for association to all probes located within 250 kb of the most proximal and distal SNPs for the associated region, allowing each probe to be tested with all SNPs in the entire LD block of the locus (166 probes in total). We determined (using SimpleM) that 1,698 effective tests were performed, which after applying a modified Bonferroni-correction [15] to account for correlation between the expression values resulted in a multiple-comparison adjusted significance cutoff of 6.7×10^−5^.

**Figure 1:**
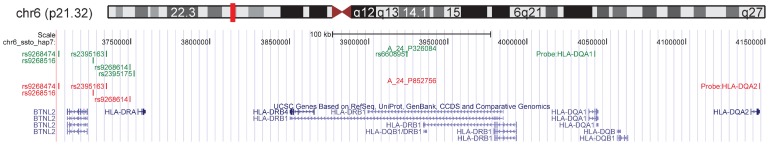
HLA region probes and SNPs involved in significant *cis* eSNP relationships. Two probes in the HLA region on chromosme 6 showing significant association with PD risk SNPs are shown on the alternative sequence haplotype chr6_ssto_hap7. Six PD risk SNPs (shown in green) showed significant association to A_24_P326084 located in *HLA-DQA1*, while four SNPs (shown in red) had significant association to A_24P852756, located in *HLA-DQA2*.

**Figure 2:**
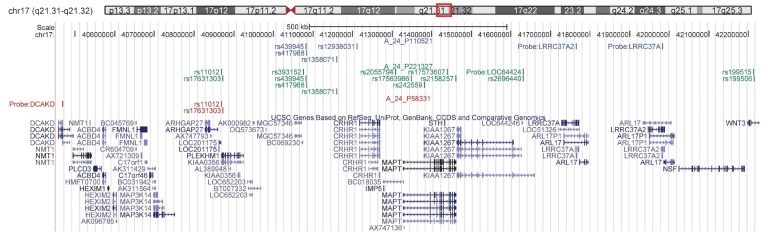
*MAPT* region probes and SNPs involved in significant *cis* eSNP relationships. Three probes in the *MAPT* region on chromosme 17 showing significant association with PD risk SNPs are displayed. Significant SNP associations with A_24_P110521 located in the duplicated genes *LRRC37A* and *LRRC37A2* are shown in blue, while SNPs associated with A_24_P221327 located in *LOC644246* are shown in green and SNPs associated with A_24_P58331 (in *DCAKD*) are shown in red.

Thirty-one SNP-probe associations reaching this adjusted level for statistical significance were observed. These associations included five probes in two regions, the HLA locus on chromosome 6 (two probes, Figure 1) and the *MAPT* region on chromosome 17 (3 probes, Figure 2).

The strongest SNP association for each of the five probes is shown in Table 2. Of these five, the strongest association was between the SNP rs2395163 (located 20 kb 5′ of *HLA-DRA*) and a probe targeting the gene *HLA-DQA1* (p = 2.2e-9). The same SNP also showed significant association to a probe in *HLA-DQA2* (p = 5.1e-7). These two probes showed moderate correlation in their expression levels (R^2^ = 0.42, p<0.0007). In stratified analyses, similar associations to increased expression levels were observed in both PD cases and controls for all SNP-probe combinations.

**Table 2:** *Cis*-Acting SNP effects on gene expression for probes within 250 kb of the PD associated risk loci.

Chr	Region	Top SNP	Minor Allele	PD-GWAS meta analysis		Agilent Probe ID	Probe Gene	Total number significant SNPs	eSNP 2df p-value	PD Cases		Controls	
				Risk OR	p-value					Effect estimate	p-value	Effect estimate	p-value
6	HLA	rs2395163	G	0.81	2.6E-11	A_24_P326084	*HLA-DQA1*	6	2.2E-09	1.06	6.7E-04	1.28	2.7E-04
						A_24_P852756	*HLA-DQA2*	5	5.1E-07	0.86	4.4E-03	0.89	1.1E-03
17	*MAPT*	rs439945	A	0.80	9.7E-14	A_24_P110521	*LRRC37A* *LRRC37A2*	4	2.4E-05	0.62	1.8E-02	0.74	3.8E-03
		rs199515	C	0.76	3.4E-17	A_24_P221327	*LOC644246*	14	3.1E-09	1.35	9.2E-04	1.38	1.1E-04
		rs11012	A	0.76	4.8E-16	A_24_P58331	*DCAKD*	2	3.7E-05	−0.56	1.2E-02	0.39	2.9E-03

Several SNPs in the *MAPT* region of chromosome 17 (Figure 2) showed significant association to the expression of multiple probes in that region. The expression of the probe targeting the gene *LOC644246* and the expression of the single probe targeting the duplicated genes leucine rich repeat containing 37A (*LRRC37A*) and leucine rich repeat containing 37, member A2 (*LRRC37A2*) were strongly correlated with each other (R^2^ = 0.56, p<0.0001) and were associated with several of the same SNPs (Figure 2). In the stratified analyses, an association to increased expression was observed in both cases and controls for these two probes (Table 2).

A third probe in the *MAPT* region was also observed to be significantly associated with two H2 haplotype tagging SNPs (rs11012 and rs17631303, LD R^2^ = 1 in this sample). The SNP association to the expression of this probe, targeting the gene dephospho-CoA kinase domain containing *(DCAKD)*, showed an opposite direction of effect in the PD cases versus control samples, with association to decreased expression in the PD cases (effect estimate = −0.56, p = 0.012) and increased expression in the controls (effect estimate = 0.39, p = 0.0029). This was the only *cis*-acting SNP association to show opposite effects in PD cases versus controls.

### *Trans*-Acting Effects

As a secondary hypothesis, we expanded our analyses to investigate *trans*-acting effects of the SNPs on all 39,122 probes on the microarray. To account for multiple comparisons in the *trans*-effect analysis we simply multiplied the effective number of SNPs determined by SimpleM (N = 45) by the total number of probes (N = 39,122) resulting in a Bonferroni corrected alpha level of 2.8×10^−8^.

Examination of *trans-*acting effects revealed an additional twenty-three SNP-probe associations reaching Bonferroni-adjusted statistical significance (p<2.8×10^−8^), sixteen involving SNPs from the *SNCA* region, six from the *RIT2* locus and one from the *MAPT* locus. Table 3 shows the most strongly associated SNP from each genomic region for each of the thirteen probes for which a significant association was observed.

**Table 3:** *Trans*-Acting SNP effects on gene expression.

SNP Chr	SNP Region	Top SNP	Minor Allele	PD-GWAS meta-analysis		Agilent Probe ID	Probe Gene	Probe Chr	Total Number significant SNPs	eSNP 2df p-value	PD Cases		Controls	
				Risk OR	p-value						Effect estimate	p-value	Effect estimate	p-value
4	*SNCA*	rs168552	G	1.28	2.1E-19	A_24_P280390	*RNF215*	22	2	1.9E-09	0.43	3.8E-06	0.07	3.2E-01
						A_24_P911678	*PDE5A*	4	2	2.5E-09	0.51	3.3E-05	0.14	9.2E-02
						A_23_P214743	*SIM1*	6	2	2.5E-08	0.31	3.8E-05	0.06	2.5 E-01
		rs2583975	A	0.79	5.2E-13	A_32_P202214	*LY6K*	8	2	8.3E-09	0.55	4.6E-05	0.12	1.6E-01
		rs2619360	A	1.22	1.25E-11	A_23_P212554	*TBL1XR1*	3	2	1.6E-08	0.65	2.9E-05	0.06	5.8E-01
		rs1903575	A	1.20	9.8E-06	A_23_P118493	*TOM1L1*	17	6	9.7E-11	0.86	4.4E-05	−0.03	7.4E-01
17	*MAPT*	rs2435200	A	0.86	8.72E-09	A_32_P10894	*LOC145783*	15	1	2.1E-08	0.34	1.1E-03	−0.61	3.8E-04
18	*RIT2*	rs9948019	G	1.19	4.5E-07	A_24_P651859	*AK021480*	1	1	1.1E-10	0.30	1.0E-01	−1.45	3.8E-05
						A_24_P919370	*PPARA*	22	1	3.2E-09	0.21	1.7E-01	−1.05	1.8E-05
						A_24_P341897	*ACVR1B*	12	1	4.2E-09	0.24	1.2E-01	−0.81	5.5E-06
						A_32_P220625	*THC2654007*	2	1	6.3E-09	0.06	6.9E-01	−0.94	2.6E-06
						A_24_P915294	*AL050000*	10	1	1.4E-08	0.26	1.7E-01	−1.21	8.6E-05
						A_23_P24469	*CSRP3*	11	1	2.3E-08	0.1955	0.3215	−1.203	1.9E-05

The strongest observed association was between the SNP rs1903575 (located intronic of the gene *KIAA1680*, ∼455 kb distal to *SNCA*) and a probe targeting the gene target of myb1 (chicken)-like 1 *(TOM1L1)* on chromosome 17. Five other SNPs in LD (mean R^2^ = 0.47) with rs1903575 (including two intronic *SNCA* SNPs rs3775433 and rs9995651) also showed significant association to this probe.

Another SNP in the *SNCA* region, rs168552, showed significant association to two probes: one for ring finger protein 215 (*RNF215*) on chromosome 22, and the second for phosphodiesterase 5A, cGMP-specific (*PDE5A*), on chromosome 4, 30 Mb away from the *SNCA* region. Independent associations were also observed for two other intronic *SNCA* SNPs. These observed associations involved rs2583975 and a probe targeting lymphocyte antigen 6 complex, locus K *(LY6K)* on chromosome 8, and rs2619360 and a probe targeting transducin (beta)-like 1 X-linked receptor 1 *(TBL1XR1)* on chromosome 3.

One SNP located in the first intron of *RIT2* showed significant association to six different probes (See Table 2), many of which are not well characterized.

## Discussion

This study represents the first comprehensive evaluation of all of the top PD-GWAS implicated SNPs on genome-wide transcription. In our analysis of the effects of SNPs on the expression of nearby probes (within 250 kb of the either end of the full region of the risk locus), we identified genes at both the *MAPT* and HLA loci whose expression is significantly associated with the PD GWAS SNPs. These findings provide novel insight into the genes that are potentially responsible for influencing PD risk at these loci.

It is particularly difficult to interpret the PD GWAS findings for the *MAPT* locus, which is the second most strongly implicated region in the US Parkinson Disease GWAS Consortium [10]. The region is complicated by the large and well characterized inversion which distinguishes the common “H1” haplotype associated with increased PD risk from the less common and PD-protective “H2” haplotype. The inverted region spans approximately 1.5 Mb [12] and contains more than twenty transcribed genes. Finally, the inversion creates an extraordinarily long stretch of high LD, which impedes the identification of the specific gene(s) responsible for PD risk.

Our studies reveal several important findings for the *MAPT* region. Of particular interest is the association between the PD SNPs and two probes in a duplicated region distal to *MAPT*. Little is known about the more strongly associated probe, targeting the hypothetical protein gene *LOC644246* (located within an intron of *KIAA1267*). However, previous studies [4] examining association between PD associated SNPs and expression of nearby genes observed a strong association between the SNP rs393152 (in strong LD with rs439945, R^2^ = 0.87) and *LRRC37A* in 133 neurologically normal frontal cortex samples. In addition, a genomewide study of expression and methylation QTLs in 143 normal cortical samples showed significant association between rs439945 and *LRRC37A* (p = 1.32×10^−13^) [16]. We saw no significant association between the SNPs in the region and the expression of *MAPT* itself. While it is notable that other genes showed a stronger effect than *MAPT*, limitations in power due to the size of the study prevent us from actually ruling out potential associations to any genes in the region.

Finally, a probe targeting the gene *DCAKD* (43,100,706–43,112,509) located 800 kb proximal to *MAPT* (43,971,750–44,105,697) showed an interesting pattern of association with the PD risk SNPs. In this case, PD samples with the risk allele (the major allele in this case) showed increased expression of this gene, while controls with the PD risk allele showed decreased expression. This finding was unique among the *cis*-effects in the observation of opposite effects of the SNP on probe expression, and highlights the need for studies of affected samples to identify important disease specific associations, which would go unobserved in healthy samples. Although little is known about the *DCAKD* gene, its kinase function and the disease-specific nature of the association may indicate the greatest potential for translational impact among the *MAPT* region findings.

In the HLA region, while the associated SNPs (rs2395163 at 32,387,809 showed the strongest association to gene expression and had the strongest association to PD in the region in the original meta-analysis) are located near the *HLA-DRA* gene, altered expression is seen for the genes *HLA-DQA1* (at 32,605,183–32,611,428) and *HLA-DQA2* (at 32,709,163–32,715,219), located 217 to 321 kb away. While it is not unusual to see regulatory effects which are fairly distant from the gene [See 17 for review], these findings emphasize the difficulty inherent in determining which gene is associated with disease risk in gene dense GWAS implicated regions. Although this eQTL relationship has not been previously reported in any studies of cortical samples, examination of the University of Chicago's expression quantitative trait loci data repository (http://eqtl.uchicago.edu/cgi-bin/gbrowse/eqtl/) indicated that association between rs2395163 and *HLA-DQA1* and *HLA-DQA2* has also been observed in LCLs [18]–[20] and monocytes [21].

In addition to our evaluation of *cis*-acting SNP effects, we also examined *trans*-acting SNP effects across the entire transcriptome. We observed significant associations between PD risk SNPs in the *SNCA* region and several genes across the genome. In each case, the significant association was driven by a strong increase in gene expression in the PD cases, with little to no effect observed in the controls. These results suggest that the genes implicated by this eQTL study may be involved downstream in a disease pathway initiated by *SNCA*. Interestingly, of the 18 probes showing significant modification by PD risk SNPs, the probe targeting *LY6K* (associated with the *SNCA* SNP rs2583975) was the only probe to also show significant (after Bonferroni correction) differential expression between cases and controls (p = 0.001, Table S1).

In contrast to the pattern of increased expression for probes associated with *SNCA* SNPs, the *trans* effects observed for the SNP in the *RIT2* region showed decreased expression in the control samples and little to no effect in the PD cases. While the immediate significance of this pattern is not clear, it may indicate a mechanism in which the regulation of these genes is disrupted in PD.

One limitation to using microarray data for this study is the potential for SNPs within the probe sequence to lead to false positive results in *cis* analyses. We examined the 5 probes showing significant *cis*-associations and identified two, A_24_P852756 (*HLA-DQA2*) and A_24_P58331 (*DCAKD*), with known SNPs located in the targeted region. A_24_P852756 had two known SNPs within the microarray probe, one of which was rare, but the other (rs9276439) had a MAF of 11.6% and therefore may have been present in our sample. Unfortunately, we do not have genotyping of this SNP (rs9276439) available in the microarray samples, but were able to determine that there is no LD (r^2^ = 0.012) between this SNP and the identified eSNP rs2395163 in HapMap samples, suggesting that the SNP in the probe is not driving the eSNP association.

In the A_24_ P58331 probe (*DCAKD*), there were 4 known rare SNPs with estimated minor allele frequencies ranging from 0.02% to 1.4%. In this case we were able to directly assess the sequence of the probe in 39 of the 50 microarray samples (17 cases/22 controls) and, as expected based on the allele frequencies, none of the SNPs (nor any novel variants) was observed, suggesting that within-probe SNPs are unlikely to be driving the association. However, we can certainly not rule out the possibility that additional known or unknown variants in the other associated probes may have either contributed to or masked SNP associations. This study is also limited to only those probes that were included on the used microarray and is, therefore, unable to evaluate all genes and differentiate all paralogs of transcripts of potential interest.

It should also be noted that this study is not intended to indicate an absence of either *cis*- or *trans*-acting effects of any of these SNPs on specific probes, a task which is more suited to larger control samples. Our goal is instead to identify the associations between SNPs and gene expression, which may be most relevant to PD by focusing on disease specific tissue (for which no larger arrayed PD sample exists) and expression relationships.

These studies suggest that, at the *MAPT* locus, the genes *LOC644246*, *LRRC37A* and its duplicate *LRRC37A2*, and the *DCAKD* gene may influence PD risk. It cannot be ruled out that more than one of these genes at the *MAPT* locus influence PD risk. In addition, these studies suggest that either *HLA-DQA1* or *HLA-DQA2*, or both, at the HLA locus may influence PD risk. Either sequencing studies or functional knock-down studies of these genes may lend further insights into whether mutations in them or variants that influence their expression may influence PD risk and whether they may be therapeutic targets for PD treatments.

## Materials and Methods

### Sample Selection

As described previously [13], brain tissue from the frontal cortex Brodmann area 9 (BA9) was obtained from three different brain banks: the Harvard Brain Tissue Resource Center McLean Hospital, Belmont, Massachusetts, the Human Brain and Spinal Fluid Resource Center VA West Los Angeles Healthcare Center, California, and the Banner Sun Health Research Institute, Sun City, Arizona. A subset of 33 Parkinson disease (PD) and 29 control samples were selected for a microarray study from the available pool of 118 PD and 87 control brains. We selected samples for inclusion in the microarray by the following four criteria: (1) absence of Alzheimer disease pathology (specified by neuropathological reports), (2) tissue pH>6.25, (3) similar ages of death for PD cases and controls, and (4) male gender. Tissue from BA9 was selected for study in the microarray in order to overcome limitations associated with the use of severely disease affected tissues when looking for disease specific changes, particularly when studying whole tissue homogenate samples. While the *substantia nigra pars compacta* (SN) is the brain region most involved in PD, it is almost completely depleted of dopaminergic neurons by the time of autopsy [22], whereas prefrontal cortex tissue does not show such dramatic neuronal death. Nevertheless, prefrontal cortex is very frequently neuropathologically involved in PD (Beach et al. report 74% of the cases present with Lewy bodies and associated fibers in this brain region [23]), and shows biochemical alterations related to the disease process [24], [25]. Since the Lewy bodies and associated fibers appear later during the disease in the prefrontal cortex [22], studying BA9 not only minimizes the confounding effects of loss of target cells possible in studies of the SN, it has the potential for identifying earlier more etiologically relevant disease changes, as opposed to late-stage markers of disease progression.

### Microarray Expression Study

Total RNA for the 33 PD and 29 control samples was extracted with TRIzol (Invitrogen, Carlsbad, CA). RNA was purified using the RNeasy MinElute Cleanup columns (Qiagen Sciences Inc, Germantown, MD) and quality was assayed by using the Agilent 2100 bioanalyzer with Agilent RNA 6000 Nano Assay kit. 2 µg of each RNA sample were labeled and hybridized on the same day to the One-Color Agilent 60-mer Whole Human Genome Microarray (#G4112A) at the Agilent Microarray Facility of the Whitehead Institute for Biomedical Research (Cambridge, MA). The dye-normalized and post surrogate processed signal for the green chanel, gProcessedSignal, obtained from Agilent's Feature Extraction Software was used for downstream analyses. The raw expression data for the 62 samples were evaluated for individual array quality (MA plots), array intensity distributions (boxplots and density plots) and between array comparison (heatmaps representing the distance between arrays) using the arrayQualityMetrics Bioconductor package. Nine outlier samples were detected based on the arrayQualityMetrics default criteria [26] and were removed, leaving 27 PD and 26 control samples for further analysis.

Microarray probes were removed if they had expression values outside the detectable spike-in range in more than 50% of the control arrays and more than 50% of the PD arrays, or if they had any of the Agilent flags IsWellAboveBG = 0, gIsSaturated = 1, gIsFeatPopnOL = 1, gIsFeatNonUnifOL = 1 in more than 75% of the arrays. The median expression value was used for replicated probes that passed the above filtering criteria. A total of 39,122 probes out of the total 45,015 probes present on the microarray chips were analyzed in the eSNP study. The expression data for the retained probes of the 53 arrays were quantile normalized, and the obtained values were log 2 transformed. All the microarray processing analyses were performed in R (http://www.R-project.org), using the Afi4x44PreProcess Bioconductor package. The microarray expression data for the samples that passed quality control are available as the E-MTAB-812 set in the ArrayExpress database (www.ebi.ac.uk/arrayexpress/).

### Genotyping

All 53 samples that passed the microarray quality control were included among 5,849 PD cases and controls genotyped in the US PD-GWAS Consortium meta-analysis replication sample [10]. As described in the consortium study, the samples were genotyped at the Center for Inherited Disease Research (CIDR) using a custom Illumina genotyping array of 768 SNPs. This set of SNPs included all of the top priority association results from the consortium discovery meta-analysis, as well as 109 markers for sex (N = 9) and ancestry assessment (N = 100). 63 of the 768 SNPs included on the custom array failed genotyping and were not released. Three of the 53 brain samples failed to genotype at the accepted 98% success rate and were removed from the eSNP analysis leaving 50 samples for analysis (Table 1).

### eSNP Selection

From the 705 released SNPs, an additional 18 SNPs failed quality assessment (MAF<1%, deviation for HWE p<0.001, differential missingness by case/control status or by genotype p<0.0001, or atypical clustering). Six regions (*SNCA*, *MAPT*, *GAK*, *GBA*, HLA, and *RIT2*) were identified to show genome-wide significant association to PD [10] and we, therefore, limited our analyses to SNPs located in these regions. We selected for evaluation as eSNPs all SNPs from these six regions that showed association to PD at a nominal level of significance (p<0.05) in the replication sample and had the same direction of effect as that reported in the discovery sample [10]. We further excluded SNPs with MAF less than 0.1 in the microarray samples, in order to avoid increased type-I error, which may occur with quantitative traits due to the small sample size. We identified a final set of 67 SNPs, from five different loci (all SNPs genotyped for *GBA* were known coding mutations and these were removed because of low MAF). Several loci, including the *SNCA* and *GAK* regions on chromosome 4, and the *MAPT* region on chromosome 17, included many SNPs in strong to moderate linkage disequilibrium (LD).

### Statistical Analyses

Association between the SNPs and expression levels was evaluated in the 26 PD cases and 24 controls using a 2-degree of freedom (df) linear regression model implemented in Plink [27]. The 2-df model permits a simultaneous test of association between genotype and expression and difference in association between cases and controls. This method has been used previously for eQTL studies involving mixed case and control samples and increases the power to detect effects of SNPs on expression levels that may be unique to disease [28].

In addition to including SNP, case status and the SNP×case status interaction term, the models were adjusted for RNA integrity number (RIN), postmortem interval, and age at death. We did not include sample pH due to the high correlation between pH and RIN and the greater range in values for RIN. Due to low frequency of minor allele homozygotes for many SNPs for either case or control samples, all SNP genotypes were coded and evaluated as a dominant model to avoid increased type 1 error.

### Correction for Multiple Testing

In order to account for the multiple comparisons, while recognizing the correlation between both the SNPs and the probes, a two-step approach was used to adjust the alpha levels. For the cis-analyses, the program SimpleM was used to determine the effective number of independent tests at each locus after accounting for LD [29]. By multiplying the number of probes by the effective number of SNPs at each locus, and summing across the five loci, we determined that 1,698 effective tests were performed for the *cis*-effects analysis. We used this number to apply a modified Bonferroni-correction [15] that accounts for correlation between the expression values used as the outcome variables (mean R^2^ = 0.11). This resulted in the multiple-comparison adjusted alpha level of 6.7×10^−5^.

To account for multiple comparisons in the *trans*–effect analysis on all probes on the microarray we simply multiplied the effective number of SNPs determined by SimpleM (N = 45) by the total number of probes (N = 39,122) resulting in a Bonferroni corrected alpha level of 2.8×10^−8^.

## Supporting Information

Table S1 Microarray study case/control differential expression results for probes involved in significant eSNP relationships.
(DOCX)
