# Supplementary material for: Evaluation of Parkinson Disease Risk Variants as Expression-QTLs
Source: PLoS One. 2012 Oct 5;7(10):e46199. doi: 10.1371/journal.pone.0046199 (PMC3465315; doi:10.1371/journal.pone.0046199)
Supplement: Table S1 — Microarray study case/control differential expression results for probes involved in significant eSNP relationships. (DOCX) [file pone.0046199.s001.docx]

Table S1: Microarray study case/control differential expression results for probes involved in significant eSNP relationships.

| Agilent Probe ID | Probe Gene | Chr | Probe Start | Microarray  p-value | Microarray effect estimate |
| --- | --- | --- | --- | --- | --- |
| A_24_P651859 | *AK021480* | 1 | 168167038 | 0.287 | 0.199 |
| A_32_P220625 | *THC2654007* | 2 | 64734902 | 0.726 | 0.043 |
| A_23_P212554 | *TBL1XR1* | 3 | 178222180 | 0.026 | -0.241 |
| A_24_P911678 | *PDE5A* | 4 | 120767795 | 0.780 | -0.023 |
| A_24_P852756 | *HLA-DQA2* | 6 | 32822575 | 0.067 | 0.361 |
| A_23_P214743 | *SIM1* | 6 | 100943540 | 0.035 | 0.112 |
| A_32_P202214 | *LY6K* | 8 | 143781706 | 0.001 | -0.305 |
| A_24_P915294 | *AL050000* | 10 | 114574690 | 0.132 | 0.246 |
| A_23_P24469 | *CSRP3* | 11 | 19160685 | 0.156 | 0.237 |
| A_24_P341897 | *ACVR1B* | 12 | 50674874 | 0.894 | 0.015 |
| A_32_P10894 | *LOC145783* | 15 | 54996656 | 0.231 | -0.118 |
| A_24_P58331 | *DCAKD* | 17 | 40467155 | 0.056 | -0.233 |
| A_24_P221327 | *LOC64424* | 17 | 41629713 | 0.420 | -0.219 |
| A_24_P110521 | *LRRC37A*  *LRRC37A2* | 17 | 41765074  41982491 | 0.150 | -0.275 |
| A_23_P118493 | *TOM1L1* | 17 | 50393842 | 0.874 | 0.017 |
| A_24_P280390 | *RNF215* | 22 | 29111957 | 0.957 | -0.004 |
| A_24_P919370 | *PPARA* | 22 | 45010510 | 0.219 | 0.193 |
| A_24_P326084 | *HLA-DQA1* | 6** | 4042591** | 0.801 | -0.065 |

** position is on alternative sequence haplotype chr6_ssto_hap7
